# A Case of Aortic Strain due to Spring Back Force by Stent Graft 3 Years after Thoracic Endovascular Aortic Repair

**DOI:** 10.3400/avd.cr.23-00081

**Published:** 2024-02-23

**Authors:** Kuntae Ahn, Nobuyuki Yoshitani, Hironobu Sugiyama, Takuya Misato, Taro Hayashi, Masato Yamaguchi

**Affiliations:** 1 Department of Cardiovascular Surgery, Akashi Medical Center, Akashi, Hyogo, Japan; 2 Department of Diagnostic and Interventional Radiology, Kobe University Hospital, Kobe, Hyogo, Japan

**Keywords:** TEVAR, complications after TEVAR, spring back force, distal SINE

## Abstract

A 79-year-old man underwent 1-debranched thoracic endovascular aortic repair (TEVAR) for a saccular aneurysm of the distal arch of the aorta. Computed tomography performed 3 years after surgery revealed a significant displacement of the distal side of the stent graft and severe deformity due to displacement of the aorta. There were no obvious findings after aortic dissection. Endovascular treatment was selected, and surgery was performed semiemergency. Additional TEVAR was performed to restore the aortic shape and displacement to its normal position.

## Introduction

Spring back force (SBF) refers to the force that causes the stent graft to bend and return to its original straight shape.^1^^,^^2)^ When a stent graft is placed in the aortic flexure (aortic arch), the SBF puts stress on both ends of the stent graft. Herein, we present a case in which SBF severely deformed the aorta on the distal side of the stent graft and was corrected by adding a stent graft.

## Case Report

**Figure 1:**
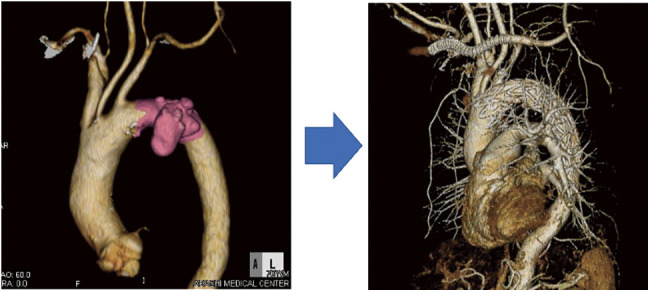
Fig. 1 1-debranched TEVAR for a distal arch aortic saccular aneurysm. TEVAR: thoracic endovascular aortic repair

**Figure 2:**
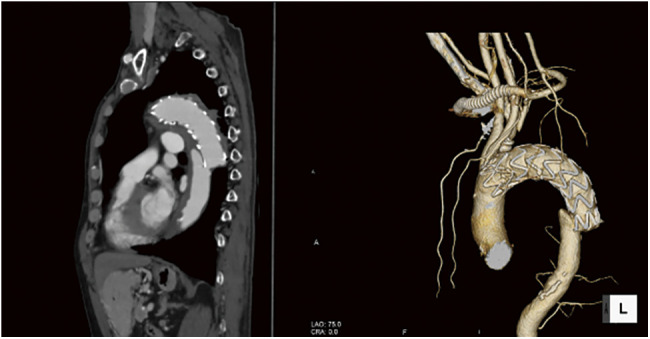
Fig. 2 CT performed at 3 years after TEVAR shows a significant deformity of the descending aorta. CT: computed tomography; TEVAR: thoracic endovascular aortic repair

Three years ago, a 79-year-old man with a history of hypertension, hyperlipidemia, and smoking (40 cigarettes a day until age 65) visited an otolaryngologist because of hoarseness and was diagnosed with left recurrent nerve palsy. Computed tomography (CT) revealed a saccular aneurysm that was laterally protruding in the distal aortic arch located 25 mm from the beginning of the left subclavian artery. A 1-debranched thoracic endovascular aortic repair (TEVAR) (axillo-axillary bypass, Valiant [Medtronic, Santa Rosa, CA, USA] 34–30 × 150 mm, Amplatzer Vascular Plug 2 [AGA Medical, Golden Valley, MN, USA] 12 × 9 mm for closure of the left subclavian artery) was performed (**Fig. 1**). The patient’s postoperative course was good. Seven days after surgery, the patient was discharged. On regular outpatient follow-up, hoarseness did not improve despite the surgery. Three years after surgery, routine CT at an outpatient clinic showed evidence of significant deviation of the distal side of the stent graft and severe deformity due to aortic displacement (**Fig. 2**).

**Figure 3:**
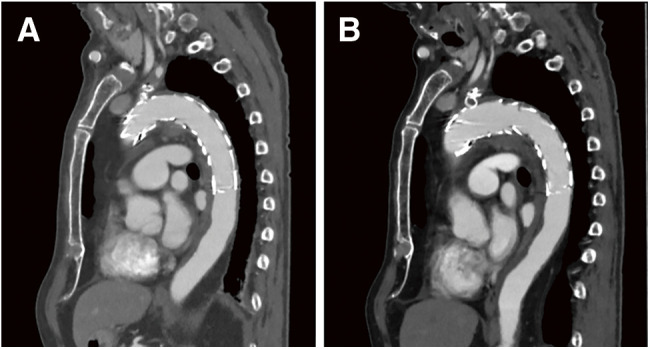
Fig. 3 (**A**) CT performed in the first postoperative year. (**B**) CT performed in the second postoperative year. No change in the shape of the descending aorta was observed; however, the curvature of the aortic arch of the stent graft increased slightly, straightening. CT: computed tomography

While reviewing images from the first and second postoperative years, no change were observed with respect to the descending aorta. However, although this was not noticed during the examination, retrospective review confirmed that the arch aortic curvature of the stent graft was slightly straightened (**Fig. 3**). The patient did not report any symptoms of chest pain or back pain, and CT did not detect obvious signs of aortic dissection or rupture. However, an urgent surgical treatment was decided due to the high possibility of aortic rupture in the future if the condition was left untreated. The preoperative ankle-brachial index was 0.9 on both sides (almost the same value as at the first surgery), and there were no findings suggesting decreased blood flow in the lower body, such as intermittent claudication or cold sense in the lower extremities. At the initial surgery, renal function was normal with an estimated glomerular filtration rate (eGFR) of 48.4, but it gradually worsened during the postoperative course, worsening to an eGFR of 34.7 at the second surgery. However, this was a gradual deterioration independent of the descending aortic deformity and was not thought to be caused by decreased renal blood flow due to the deviation of the descending aorta. Given the patient’s age at 79 years, descending aortic replacement via open left thoracotomy was considered high risk. An urgent endovascular treatment was selected instead. During surgery, an artificial heart–lung machine was placed on standby. In case of rupture, the patient was placed in a right semisupine position to allow access to the descending aorta during the left anterior lateral sternotomy and partial lower sternotomy technique.^3)^ The shape of the aorta was considered a difficulty during insertion of the wire from the distal side. Following the unlikely event that the wire could not be inserted from the lower extremity, we decided to insert the wire from the right brachial artery and guided it into the femoral artery using a Snare catheter. Therefore, the right upper extremity was thoroughly disinfected and prepared before the surgery.

**Figure 4:**
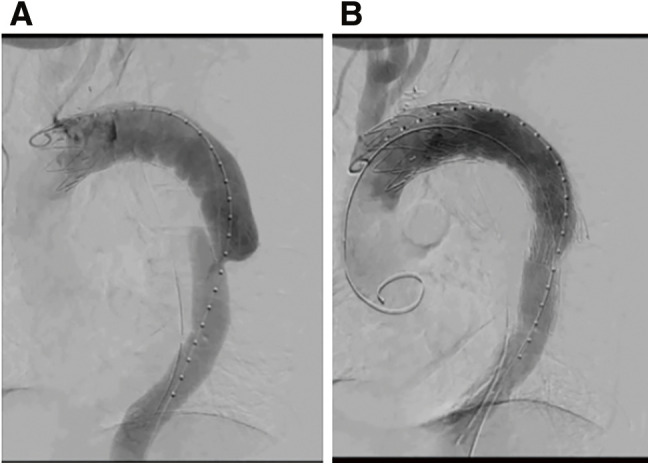
Fig. 4 Additional TEVAR was performed. (**A**) Wire was inserted from the left femoral artery and passed through the aortic deformity. (**B**) Aortic deformity was corrected by placing an additional stent graft in the aortic deformity. TEVAR: thoracic endovascular aortic repair

**Figure 5:**
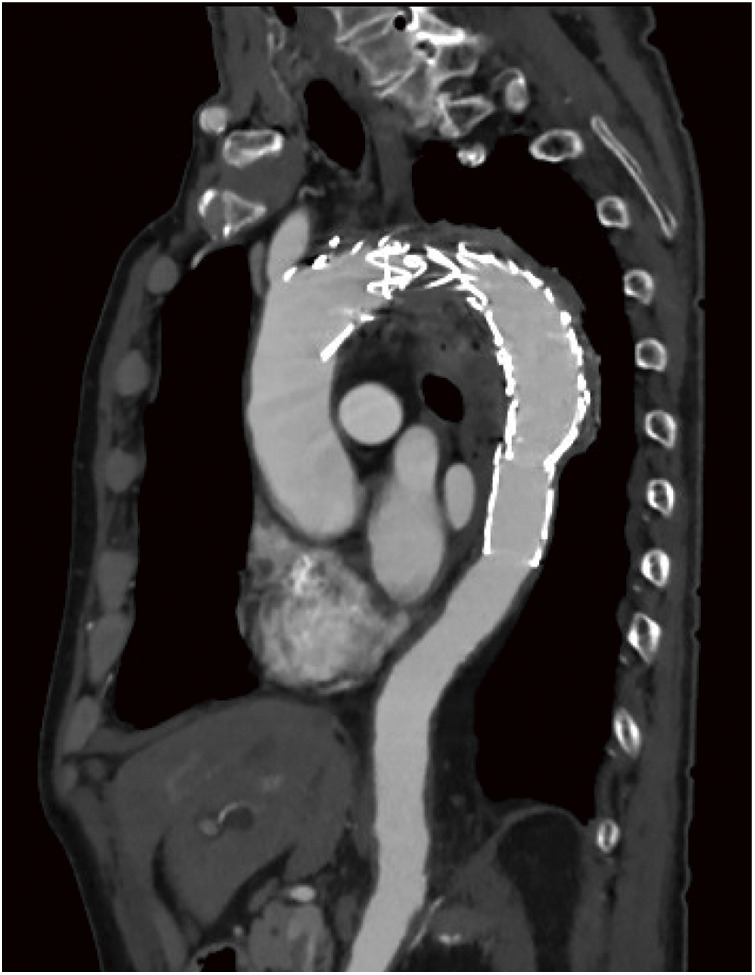
Fig. 5 Final postoperative CT image. Shape of the descending aorta was restored and there was no evidence of rupture. CT: computed tomography

An incision was made in the left inguinal region, and the approach was made through the left femoral artery. The wire was able to pass through the aortic deviation with no problem. The stent graft was landed 5 cm peripherally from the distal end of the previously implanted stent graft, and a Gore TAG (W. L. Gore & Associates, Flagstaff, AZ, USA) 31–26 × 100 mm was implanted. To stack the first implant stent graft further centrally, a Gore TAG 37–37 × 150 mm was added. After stent graft placement, ballooning was not performed to avoid strong forces on the deformed aorta. The shape of the deviated aorta was restored, and the aorta returned to its normal position (**Fig. 4**). The postoperative contrast-enhanced CT scan did not detect obvious endoleak, rupture, or distal stent graft-induced new entry tear (SINE) findings (**Fig. 5**). There were no postoperative complications such as spinal cord injury. The patient was discharged on his own on the seventh postoperative day.

## Discussion

When a stent graft is placed in the aorta, SBF occurs at the peripheral end of the stent graft in the aorta. It can cause distal SINE by imparting strong physical stress to the aortic curvature side of the aorta.^4)^ In addition to SBF, other factors that can cause distal SINE include patient- and stent graft-related factors. Patient factors include a history of aortic dissection at the site of stent graft implantation. Chronic dissection is considered to be at higher risk than acute dissection,^5)^ but this patient neither had a history of aortic dissection nor did he have a history of connective tissue abnormalities such as Marfan syndrome or Loeys–Dietz syndrome. First factor in the stent graft body is the oversizing of the stent graft relative to the aorta.^2)^ In this case, the preoperative aortic diameter of the stent graft placement was 25 mm, and the distal size of the stent graft used was 30 mm in diameter, which was considered as an appropriate size. Beyond that, SBF has been associated with several factors including stent graft material, stent skeletal design, and graft material. SBF is particularly strong when using devices with bars that longitudinally connect the stent skeleton.^4^^,^^5)^ A strong SBF is applied to the aortic wall when the length of the stent graft used is less than 145 mm,^6)^ when there is strong bending of the arch aorta,^7)^ and when the angle of the stent graft obliteration side to the aortic wall is large.^8)^ However, commercially available stent graft devices differ in material and stent shape, and each device is considered to have a different SBF. Valiant is a highly flexible and adaptable device that can easily fit various aortic configurations.^9)^ Furthermore, there is no connecting bar in Valiant. In the present case, Valiant used in the initial surgery had a length of 150 mm. The arch aorta also has a relatively gentle bend, and the implanted stent graft fits into the straight portion of the descending aorta, which is outside of the conditions reported so far in which SBF is likely to adversely affect the aorta.

In this case, imaging findings did not show obvious findings of aortic dissection in the aortic deviation. Therefore, it was speculated that the aorta was not deviated as a result of distal SINE, but the SBF might have largely deformed the aorta toward the greater curvature while maintaining its luminal structure. However, the possible occurrence of distal SINE was not completely ruled out because the aorta was not opened by open surgery and it did not undergo pathological examination. In any case, the imaging findings of a large deviation of the aorta toward the aortic arch suggest that SBF is definitely the cause of the present symptoms.

To the best of our knowledge, no case of such a strong aortic deviation has ever been reported. Treatment by artificial vascular replacement of the descending aorta through an open left thoracic approach may be appropriate. However, considering operative tolerance, endovascular treatment according to the distal SINE procedure was deemed safer for the patient considering his advanced age (79 years).

In this case, the patient was asymptomatic despite the large deviation in the aorta. Despite this, the CT follow-up showed that the curvature of the stent graft in the arch aortic region had increased and was close to a straight line, although this was not noticed at the time of imaging, but upon retrospective confirmation (**Fig. 3**). This may have captured the gradual deformation of the stent graft according to SBF.

The occurrence of a strong aortic deviation after stent grafting, as in this case, is extremely rare. Endovascular treatment may be able to correct the deviation without necessarily performing open thoracotomy. However, when performing endovascular treatment, damage to the aorta cannot be ruled out when such a greatly deviated aorta is rapidly returned to its original state. When similar symptoms are present and endovascular treatment is selected, physicians must prepare for any rupture. If the patient has already had an aortic rupture or if it is difficult to insert a wire from either the proximal or distal arteries, open surgery, rather than endovascular treatment, should be considered as on priority.

## Conclusion

Strong deviation of the descending aorta due to SBF, which occurred in the remote phase after TEVAR surgery for a distal arch aortic aneurysm, was successfully corrected via TEVAR. In all cases of stent graft insertion, SBF may impact the aorta and cause adverse effects, including distal SINE. Therefore, periodic CT follow-up is always considered necessary after TEVAR surgery, regardless of the presence or absence of symptoms.

## Consent for Publication

Consent for this study was obtained from the patient.

## Disclosure Statement

None declared.

## Author Contributions

Study conception: KA

Writing: KA

Critical review and revision: All authors

Final approval of the article: All authors

Accountability for aspects of the work: All authors.
